# Store operated calcium entry is altered by the inhibition of receptors tyrosine kinase

**DOI:** 10.18632/oncotarget.24685

**Published:** 2018-03-23

**Authors:** Noémie Emeriau, Marie de Clippele, Philippe Gailly, Nicolas Tajeddine

**Affiliations:** ^1^ Laboratory of Cell Physiology, Institute of Neuroscience, Université catholique de Louvain, Brussels, Belgium

**Keywords:** store-operated calcium entry, EGFR, ErbB2, receptors tyrosine kinase, targeted therapies

## Abstract

SOCE (Store-Operated Calcium Entry) is the main mechanism by which external Ca^2+^ enters into non-excitable cells after endoplasmic reticulum emptying. It is implicated in several processes such as proliferation and migration. Alterations in SOCE could initiate or support the development of hallmarks of cancer. In this project, we showed that disruption of the EGFR/ErbB2-dependent signalling by lapatinib and CP-724714, two inhibitors of the receptor tyrosine kinase (RTK), dramatically reduced the amplitude of the SOCE in breast cancer cells. LY294002 and MK2206, two inhibitors of the PI3K/Akt pathway, mimicked the effect of the inhibition of EGFR/ErbB2. In contrast, inhibitors of the MAPK pathway had no effect on SOCE. The involvement of EGFR/ErbB2 receptors and the PI3K/Akt pathway in the regulation of SOCE was confirmed in other cell lines derived from various cancer types. All these results showed that SOCE is positively regulated by the PI3K/Akt pathway and that this effect may be suppressed by the inhibition of the upstream RTKs. Inhibition of SOCE might therefore contribute to the anticancer effects of RTK inhibitors.

## INTRODUCTION

A multitude of studies show that alterations in Ca^2+^ signalling initiate or support the development of hallmarks of cancer and Ca^2+^ channels are centrally involved in cancer cells proliferation and migration [1, 2]. In particular, the role of store-operated calcium entry (SOCE) in tumorigenesis and tumour progression has been subject to intense investigation.

Initially described by James Putney in 1986, SOCE is the main mechanism by which external Ca^2+^ enters into non-excitable cells [3]. Upon endoplasmic reticulum (ER) depletion in response to the activation of a G-protein coupled receptor or a receptor tyrosine kinase, stromal interaction molecule 1 (STIM1), an ER-resident membrane protein, clusters at the ER/plasma membrane junctional region, where it triggers the opening of ORAI1, a plasma membrane channel, which allows Ca^2+^ entry from the external medium [4–6]. It should be noticed that all stimuli that provoke ER emptying also induce SOCE. It is then possible to trigger SOCE *in vitro* even in cells for which specific agonists are unknown. For example, inhibition of SERCA pumps by thapsigargin leads to the release of Ca^2+^ from the ER and induces SOCE.

If the crucial need of STIM1 and ORAI1 for generating SOCE is largely accepted, the implication of TRP channels, especially TRPC1, is more debated [7]. In non-small cell lung carcinoma (NSCLC) cells, we observed that TRPC1 was involved in SOCE and that TRPC1 depletion altered EGFR activation and induced a G0/G1 cell cycle arrest resulting in a dramatic decrease in cell growth [8]. Other reports have shown that SOCE was involved in cancer cell proliferation. For instance, it has been shown that STIM1 depletion increased p21 expression and decreased Cdc25C expression that led to significant inhibition of cervical cancer cells proliferation [9]. More recently, it has been reported that TGF-β induced cell cycle arrest in breast cancer cells by reducing STIM1 expression and SOCE amplitude [10]. Furthermore, clinical studies have revealed an association between the expression of SOCE components and cancer prognosis. In NSCLC, high levels of ORAI1 were associated with a shorter survival [11]. ORAI1 is also overexpressed in colorectal cancers and significantly correlated with advanced clinical stage and high incidence of metastasis [12]. Similar results were obtained by investigating the impact of STIM1 in colorectal cancer outcome [13]. High expression of ORAI3, which can mediate SOCE at least in some cellular models, is associated with a poor prognosis in NSCLC [14].

Agents targeting receptor tyrosine kinases (RTKs) have become major components of the therapeutic arsenal against various malignancies, notably leukemia, NSCLC and breast cancer. Their discovery probably constitutes one of the most important advances in cancer treatment in the last two decades. Upon binding to their ligand, RTKs dimerize, resulting in autophosphorylation of their cytoplasmic domains and activation of tyrosine kinase activity. Multiple cytoplasmic signalling pathways, including the Ras/Raf mitogen-activated protein kinase pathway (MAPK), the phosphoinositol 3’-kinase/Akt pathway (PI3K/Akt), the signal transducer and activator of transcription pathway (STAT), the protein kinase C (PKC) pathway, the phospholipase C, and scaffolding proteins may then be activated [15–17]. They then modulate fundamental processes that are classically deregulated in cancer cells such as proliferation, apoptosis or migration. For the late two decades, a large variety of small molecules or antibodies have been developed to interfere with RTKs by competing with their ATP binding site or with their ligand binding site or by triggering their degradation.

ErbB2 (also called Her2/neu) is an RTK belonging to the family of ErbB receptors. Contrarily to other ErbB receptors, ErbB2 has no known ligand and acts by binding with other ErbB receptors. Amplification of ErbB2 is found in about 20% of breast cancers and is classically associated with a poor prognosis [18]. The use of monoclonal antibodies targeted against ErbB2 has completely revolutionized the treatment of advanced breast cancers overexpressing ErbB2. Trastuzumab is a humanized monoclonal antibody binding the domain IV of ErbB2. It is now largely used in the treatment of ErbB2-positive breast cancers [19]. Its mode of action remains debated but it is usually postulated that trastuzumab inhibits Her2/neu dimerization and phosphorylation [20]. Unfortunately, initial response to trastuzumab is observed in less than 35% of patients with ErbB2 positive breast cancers. Moreover, 70% of initial responders treated with trastuzumab have a disease progression within one year [21]. One of the mechanisms explaining trastuzumab resistance is the expression of a truncated form of ErbB2 (p95ErbB2) in which the binding domain of trastuzumab is lacking.

Lapatinib is a dual inhibitor of EGFR and ErbB2. Unlike trastuzumab, lapatinib targets the intracellular domain of ErbB2 by acting as an ATP competitor and is then able to inhibit p95ErbB2 [22]. In combination with the fluoropyrimidine capecitabine, lapatinib is more efficient than capecitabine alone in women with ErbB2-positive advanced breast cancer that has progressed after treatment with anthracyclines, taxanes, and trastuzumab [23, 24]. However, the effect of lapatinib on Ca^2+^ signalling in cancer cells has never been investigated.

Given the fact that SOCE is involved in cancer cell proliferation and that RTKs might be, at least in some circumstances, linked to Ca^2+^ signalling, we have evaluated the effects of tyrosine kinase inhibitors (TKIs) or monoclonal antibodies targeting RTKs in the regulation of SOCE in ErbB2-overpressing breast cancer cell line. We observed that trastuzumab had no effect on SOCE amplitude. In contrast, lapatinib dramatically decreased Ca^2+^ influx elicited by ER depletion. Our data suggest that this effect was mediated by the inhibition of PI3K/Akt signalling pathway. Modulation of SOCE might therefore constitute an alternative mechanism of action of TKIs.

## RESULTS

### Lapatinib decreased SOCE amplitude

**Figure 1:**
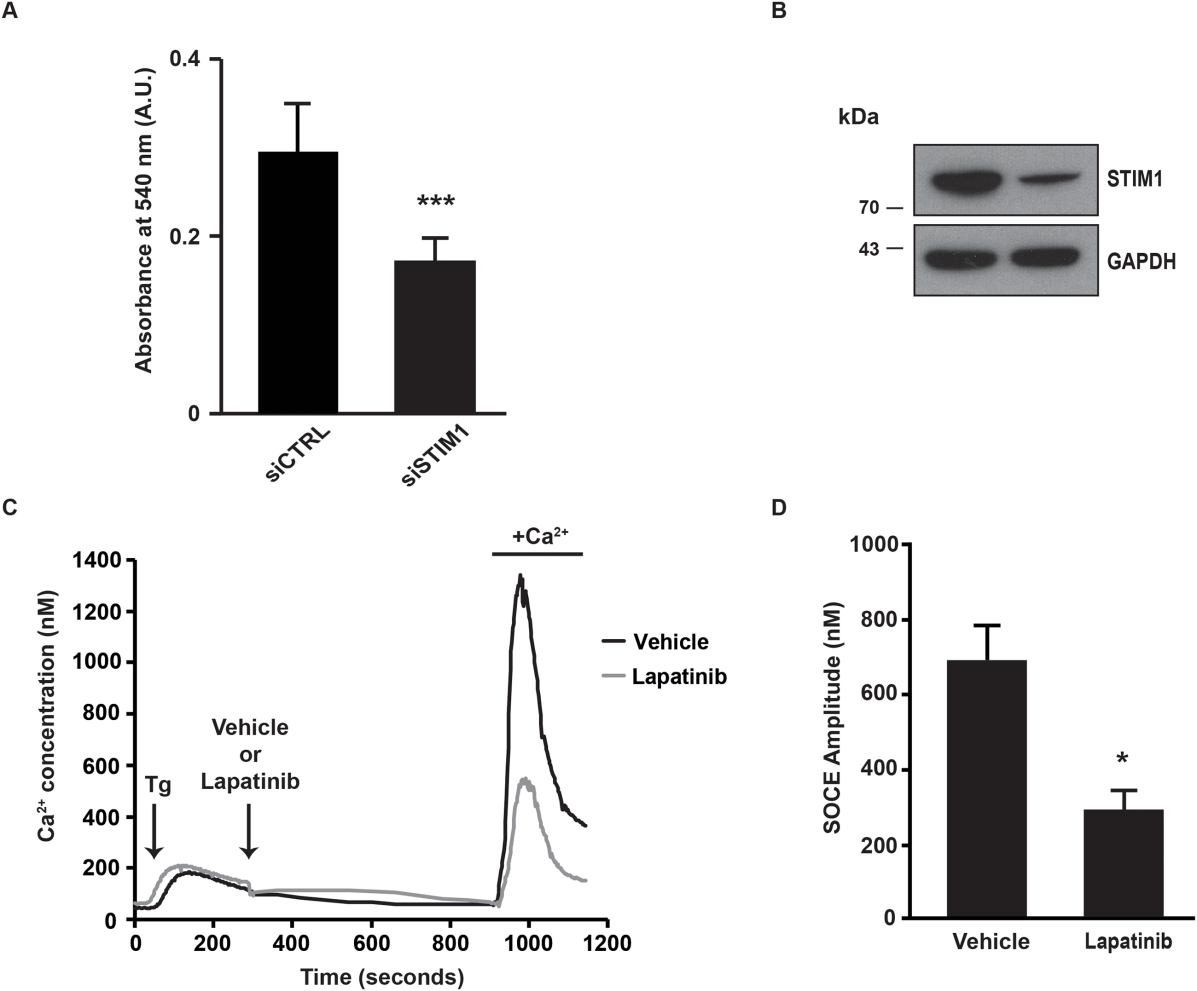
SOCE amplitude is decreased after lapatinib treatment **(A)** One day after transfection with either a pool of siRNAs downregulating STIM1 (siSTIM1) or a pool control siRNAs (siCTRL), SK-BR-3 cells were plated on 96-well plates. Four days later, cell proliferation was measured with an *in vitro* proliferation assay (sulforhodamine B based, Sigma-Aldrich). The absorbance measured at 540 nm was proportional to the cellular density. **(B)** Immunoblot analysis showing the effect of siSTIM1 transfection on STIM1 expression in comparison with siCTRL transfection. GAPDH is used as a loading control. **(C)** In order to observe only the calcium response corresponding to the entry of calcium in cells (SOCE) we used a specific protocol allowing the decoupling of endoplasmic reticulum (ER) emptying and SOCE. SOCE was triggered by Ca^2+^ depletion induced by thapsigargin (Tg). Tg was added in the absence of external Ca^2+^, in a solution of Krebs EGTA. The first Ca^2+^ peak reflects the release of Ca^2+^ from the ER. Lapatinib 2μM (or a vehicle) was then added, after 10 minutes Ca^2+^ was added in the external medium, allowing SOCE. Traces are representative of nine independent experiments (minimum 25 cells analysed per experiment). **(D)** Quantification of experiments presented in C: [Ca^2+^]_i_ measured at the peak induced by SOCE. Comparison of SK-BR-3 cells treated with vehicle or lapatinib 2μM is shown. Results are expressed as means ± S.D (n=9), Student’s t-test, ^*^ *P* < 0.05, ^**^ *P* < 0.01, ^***^ *P* < 0.001.

**Figure 2:**
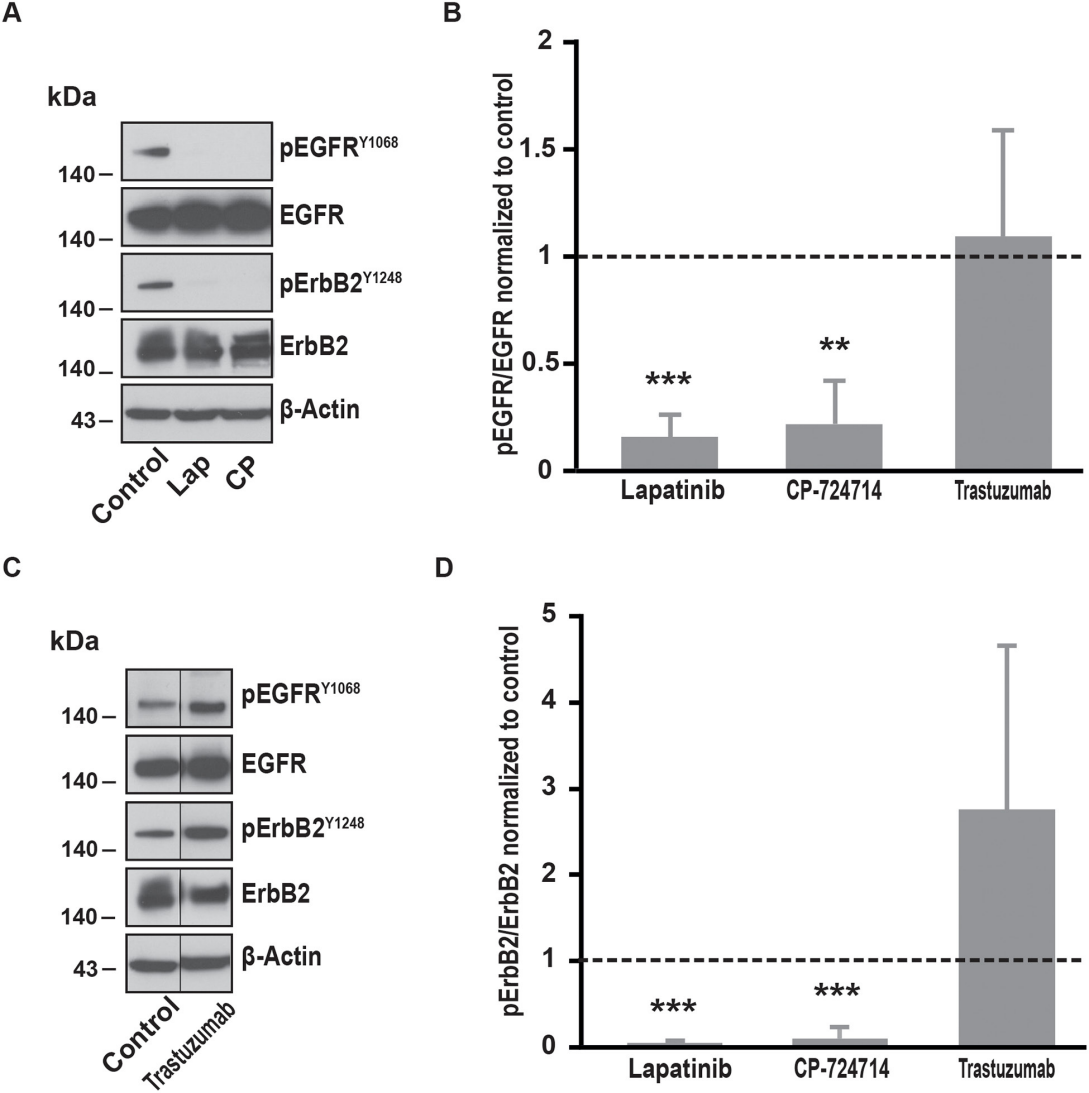
Effects of ErbB2 specific inhibitors on EGFR and ErbB2 phosphorylation **(A)** Immunoblot analysis of phospho-EGFR^Y1068^, total EGFR, phospho-ErbB2^Y1248^ and total ErbB2 in SK-BR-3 cells. Cells were starved for one hour and a half, then treated at 37°C for 10 minutes with lapatinib 2μM, CP-724714 2μM or a vehicle to reproduce Ca^2+^ measurement conditions. Immunoblots presented in A are representative of at least three independent experiments. **(B)** Relative quantification with ImageJ software of immunoblots presented in A and C. EGFR phosphorylation obtained after lapatinib 2μM, CP-724714 2μM or trastuzumab 1μM treatment was compared to total EGFR and then normalized to the control condition. Results are expressed as means ± S.D of at least three independent experiments, Student’s t-test, ^**^ *P* < 0.01, ^***^ *P* < 0.001. **(C)** Immunoblot analysis of phospho-EGFR^Y1068^, total EGFR, phospho-ErbB2^Y1248^ and total ErbB2 in SK-BR-3 cells. SK-BR-3 cells were treated in the same conditions presented in A but with tratsuzumab 1μM. Immunoblots presented in C are representative of at least three independent experiments. Lanes separated by a thin line have been cropped from the same immunoblot. **(D)** Relative quantification of immunoblots presented in A and C. ErbB2 phosphorylation obtained after lapatinib 2μM, CP-724714 2μM or trastuzumab 1μM treatment was compared to total ErbB2 and then normalized to the control condition. Results are expressed as means ± S.D of at least three independent experiments, Student’s t-test, ^***^ *P* < 0.001.

In order to evaluate the role of ErbB2 in the modulation of SOCE, we used SK-BR-3 cells that are derived from a pleural effusion of a breast cancer overexpressing ErbB2. First of all, we wanted to verify that inhibition of SOCE decreased proliferation of SK-BR-3 cells. As expected, siRNA-mediated depletion of STIM1, a major molecular player of SOCE, dramatically reduced the proliferation of SK-BR-3 cells (Figure 1A). The effect of siRNAs pool on STIM1 expression is shown in Figure 1B. For measuring the amplitude of SOCE, cells were first treated by thapsigargin in the absence of external Ca^2+^. The first peak thus corresponds to the release of Ca^2+^ from the ER (Figure 1C). Five minutes after thapsigargin stimulation, lapatinib was added for additional 10 minutes. Then, Ca^2+^ was re-introduced in the external medium. The second peak is representative of the SOCE. As shown in Figure 1C, 1D and in Table 1, lapatinib dramatically decreased the ER-dependent Ca^2+^ entry. It is to note that this effect is already present 10 minutes after the addition of lapatinib, this excluding an effect of the inhibitor on the expression of SOCE mediators (i.e. STIM1, ORAI1, TRPC1, …). In SK-BR-3 cells, lapatinib abolished EGFR and ErbB2 phosphorylation (Figure 2).

**Table 1:** Effects of various inhibitors on SOCE amplitude, expressed as percentage of control (mean ± SD)

Inhibitor	SOCE ± SD (% of control)	N	*P* value
Lapatinib 2μM	40.68 ± 26.15	9	<0.05
Trastuzumab 1μM	81 ± 32.78	10	NS
CP-724714 2μM	75.79 ± 17.86	12	<0.05
CP-724714 20μM	53.33 ± 26.58	8	<0.001
Erlotinib 5μM	159.85 ± 34.58	12	<0.001
Gefitinib 1μM	99.83 ± 42.84	10	NS
LY294002 25μM	39.41 ± 11.99	10	<0.001
MK-2206 1μM	68.21 ± 11.93	10	<0.01
Trametinib 10μM	72.06 ± 39.28	10	NS
Ruxolitinib 1μM	130.33 ± 39.54	10	NS

SOCE was measured as in Figure 1A.

### Effects of ErbB2 specific inhibitors on SOCE amplitude

Since lapatinib is a dual inhibitor of EGFR and ErbB2, we wanted to evaluate the respective involvement of both receptors in the modulation of SOCE. We then repeated the experiments described in Figure 1A by replacing lapatinib by trastuzumab. Surprisingly, trastuzumab did not alter the SOCE (Table 1). Importantly, in SK-BR-3 cells, trastuzumab was not able to interfere with ErbB2-dependent signalling. Indeed, 10 minutes treatment with trastuzumab did not modify ErbB2 phosphorylation (Figure 2). However, ErbB2 activation was reduced by longer treatments with trastuzumab (from 24h to 72; data not shown). In line with this observation, trastuzumab slightly reduced SK-BR-3 cells proliferation (see below). Altogether, those observations suggest that SK-BR-3 cells are sensitive to trastuzumab but that short exposure to this compound is not sufficient to disrupt ErbB2-dependent signalling.

In contrast, CP-724714, a specific inhibitor of the ErbB2 kinase activity that has been tested for the treatment of ErbB2-positive malignancies [25, 26], reduced SOCE amplitude (Table 1). This effect was quite modest for a concentration of 2μM CP-72714 but was dramatically improved with a ten times higher concentration. As expected, CP-724714 strongly decreased ErbB2 phosphorylation (Figure 2). Importantly, CP-724714 also diminished the phosphorylation of EGFR. This might be due to the fact that EGFR can be activated by ErbB2 since it is its main partner.

**Figure 3:**
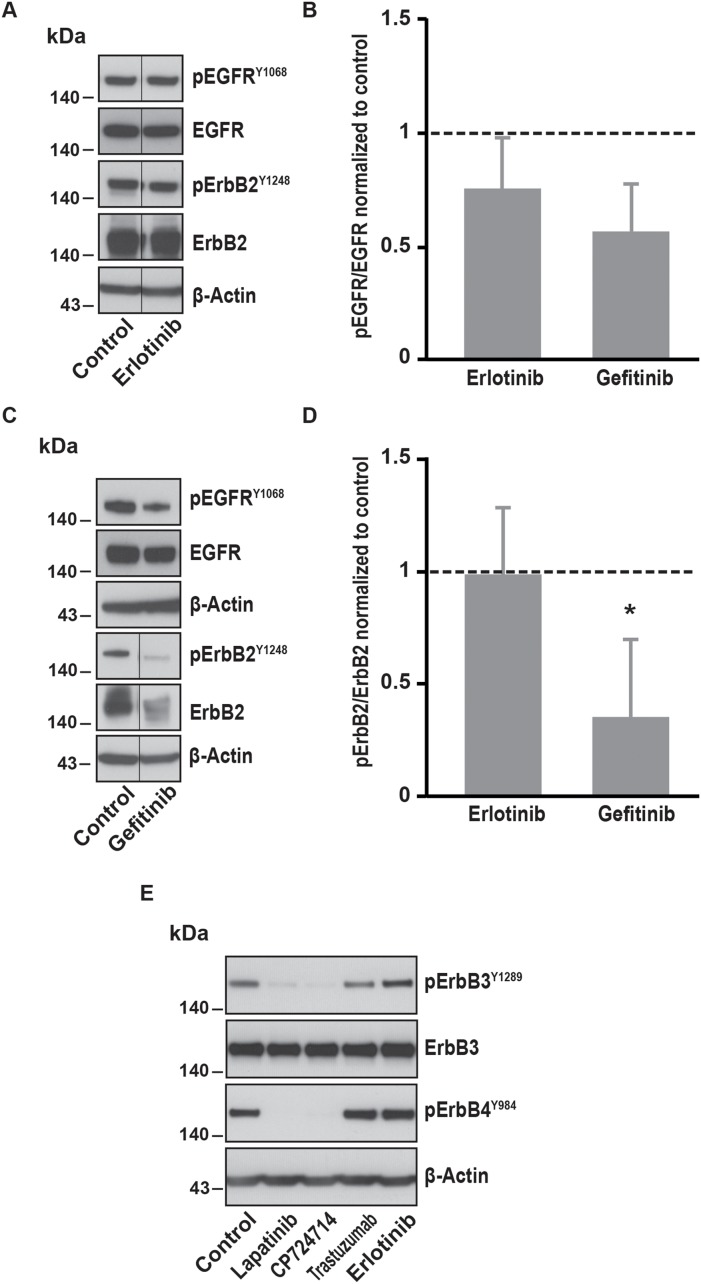
Effects of EGFR specific inhibitors on EGFR, ErbB2, ErbB3 and ErbB4 phosphorylation **(A)** Immunoblot analysis of phospho-EGFR^Y1068^, total EGFR, phospho-ErbB2^Y1248^ and total ErbB2 in SK-BR-3 cells. The same protocol than in Figure 2 was used, SK-BR-3 were treated at 37°C for 10 minutes with erlotinib 5μM or a vehicle. Immunoblots presented in A are representative of at least three independent experiments. Lanes separated by a thin line have been cropped from the same immunoblot. **(B)** Relative quantification of immunoblots presented in A and C. EGFR phosphorylation obtained after erlotinib 5μM or gefitinib 1μM treatment was compared to total EGFR and then normalized to the control condition. Results are expressed as means ± S.D. of at least three independent experiments, Student’s t-test, non significant. **(C)** Immunoblot analysis of phospho-EGFR^Y1068^, total EGFR, phospho-ErbB2^Y1248^ and total ErbB2 in SK-BR-3 cells. SK-BR-3 cells were treated 10 min with gefitinib. Immunoblots presented in C are representative of at least three independent experiments. Lanes separated by a thin line have been cropped from the same immunoblot. **(D)** Relative quantification of immunoblots presented in A and C. ErbB2 phosphorylation obtained after erlotinib 5μM or gefitinib 1μM treatment was compared to total ErbB2 and then normalized to the control condition. Results are expressed as means ± S.D of at least three independent experiments, Student’s t-test, ^*^ *P* < 0.05. **(E)** Immunoblot analysis of phospho-ErbB3^Y1289^ and phospho-ErbB4^Y984^ in SK-BR-3 cells. SK-BR-3 cells were treated 10 min with lapatinib 2μM, CP-724714 2μM, trastuzumab 1μM, erlotinib 5μM or the vehicle. Immunoblots presented in E are representative of two independent experiments. β-actin was used as a loading control.

In contrast, specific inhibitors of EGFR such as erlotinib and gefitinib were not able to decrease SOCE amplitude in SK-BR-3 cells (Table 1). Erlotinib had only slight effects on EGFR activation and no effect on ErbB2 activation (Figure 3). Gefitinib significantly inhibited ErbB2 phosphorylation but not EGFR phosphorylation. This suggests that inhibition of ErbB2 alone is not sufficient to interfere with SOCE signalling.

Since ErbB2 is known to be the preferred partner not only of the EGFR but also of the ErbB3 and ErbB4 receptors, we investigated the effect of the inhibition of ErbB2 on the activation of those receptors. We found that the phosphorylation of both ErbB3 and ErbB4 was almost abolished after lapatinib and CP-724714 treatment (Figure 3E). In contrast, trastuzumab had no effect on ErbB3 and ErbB4 activation. As expected, erlotinib was devoid of effect on those receptors. It is then possible that the effect ErbB2 inhibition on SOCE was also dependent on ErbB3 and/or ErbB4. Unfortunately, this hypothesis is challenging to investigate since specific inhibitors of ErbB3 and ErbB4 do not exist. However, neuregulin 1, which is an activator of ErbB2/ErbB3 and ErbB2/ErbB4 heterodimers, was unable to increase SOCE amplitude (data not shown). This suggests that the role of ErbB3 and ErbB4 in the modulation of SOCE is modest.

### Involvement of downstream pathways of EGFR and ErbB2 in SOCE regulation

**Figure 4:**
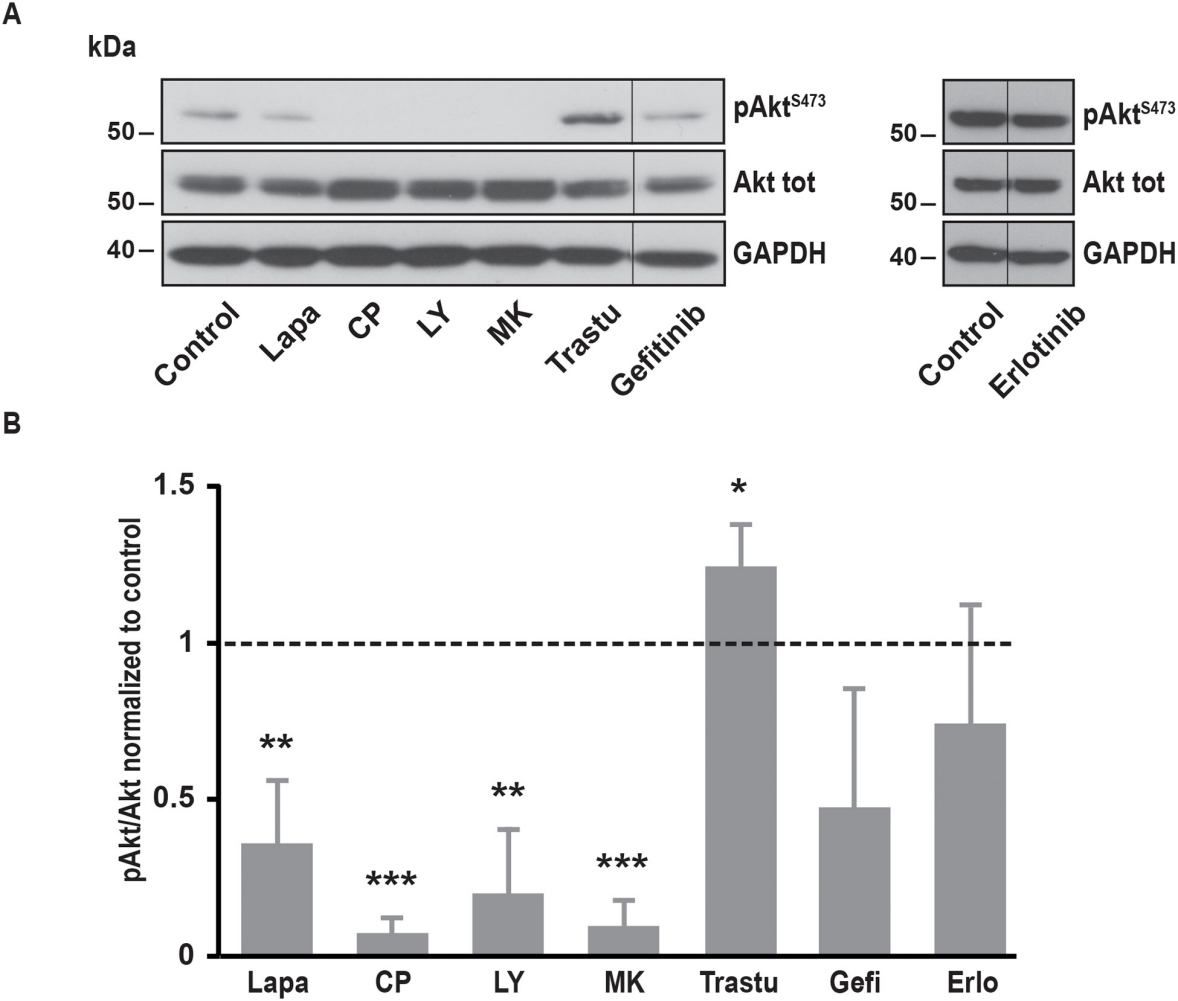
Effects of various inhibitors on Akt phosphorylation **(A)** Immunoblot analysis of phospho-Akt^S473^ and Akt total in SK-BR-3 cells. The same protocol than in Figure 2 was used, SK-BR-3 were treated at 37°C for 10 minutes with lapatinib 2μM, CP-724714 2μM, LY294002 25μM, MK-2206 1μM, trastuzumab 1μM, gefitinib 1μM, erlotinib 5μM or a vehicle. Immunoblots presented in A are representative of at least three independent experiments. Lanes separated by a thin line have been cropped from the same immunoblot. **(B)** Relative quantification of immunoblots presented in A. Akt phosphorylation obtained after treatment was compared to Akt total and then normalized to the control condition. Results are expressed as means ± S.D of at least three independent experiments, Student’s t-test, ^*^ *P* < 0.05, ^**^ *P* < 0.01, ^***^ *P* < 0.001.

**Figure 5:**
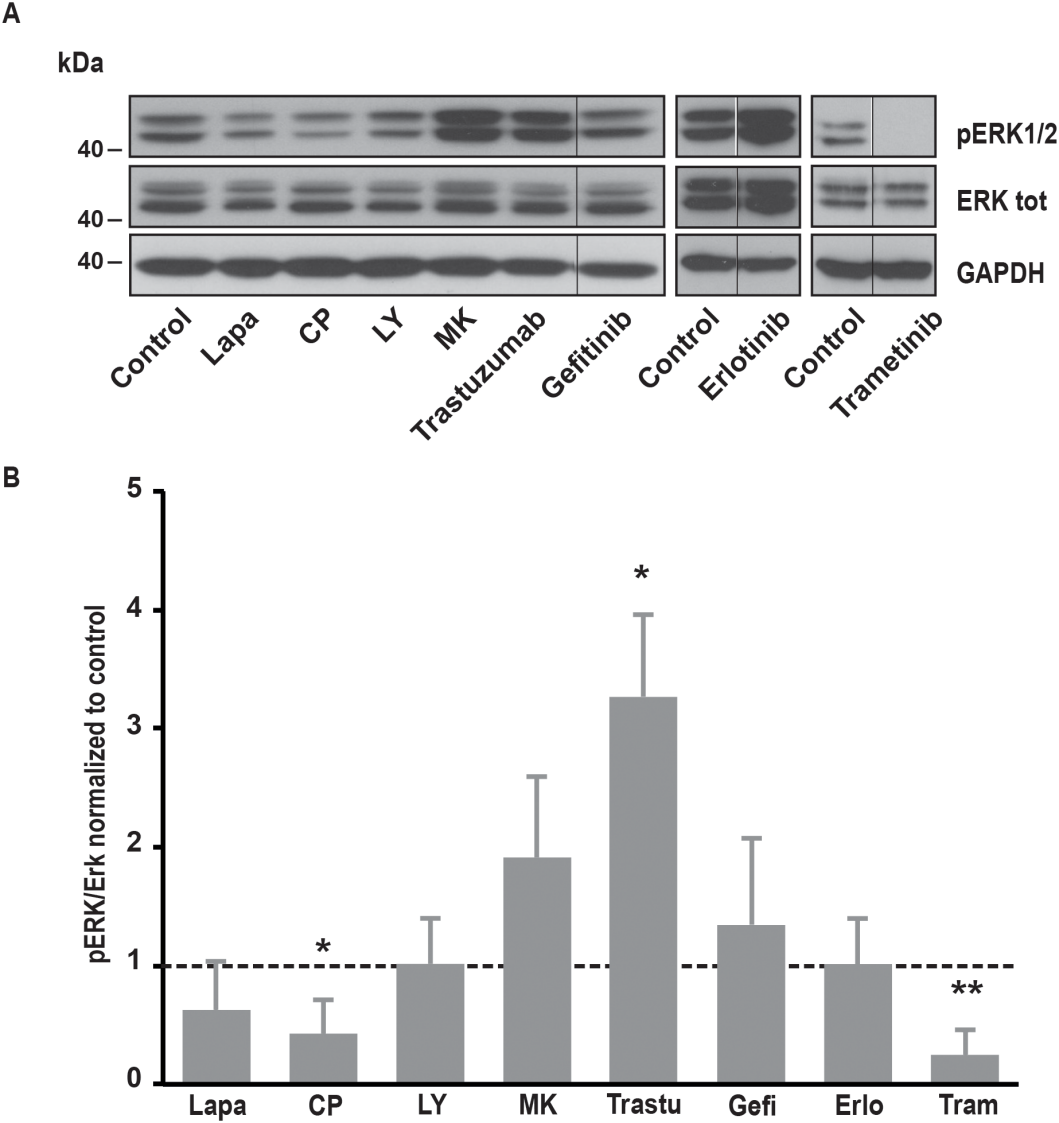
Effects of various inhibitors on ERK1/2 phosphorylation **(A)** Immunoblot analysis of phospho-ERK1/2 and ERK1/2 total in SK-BR-3 cells. The same protocol than in Figure 2 was used, SK-BR-3 were treated at 37°C for 10 minutes with lapatinib 2μM, CP-724714 2μM, LY294002 25μM, MK-2206 1μM, trastuzumab 1μM, gefitinib 1μM, erlotinib 5μM, trametinib 10μM or a vehicle. Immunoblots presented in A are representative of at least three independent experiments. Lanes separated by a thin line have been cropped from the same immunoblot. **(B)** Relative quantification of immunoblots presented in A. ERK1/2 phosphorylation obtained after treatment was compared to ERK1/2 total and then normalized to the control condition. Results are expressed as means ± S.D of at least three independent experiments, Student’s t-test, ^*^ *P* < 0.05, ^**^ *P* < 0.01.

It is well known that EGFR and ErbB2 induce the activation of several downstream pathways involved in cell proliferation, cell migration and resistance to cell death. Among them, PI3K/Akt and MAPK pathways are frequently upregulated in cancer cells. In SK-BR-3 cells, lapatinib and CP-724714 strongly reduced Akt phosphorylation on serine 473 (Figure 4). Surprisingly, lapatinib had only minor effects on ERK1/2 activation, suggesting that its impact on MAPK pathway is limited in SK-BR-3 cells (Figure 5). CP-724714 decreased ERK1/2 activation of about 50%, an effect that is considerably less important than the effect of the same compound on the PI3K/Akt pathway. In contrast, trastuzumab inhibited neither PI3K/Akt- nor the MAPK-dependent signalling (Figure 4 and 5). On the contrary, trastuzumab strongly activated ERK1/2. Similar observations have already been performed in other cell lines but the interpretation of this surprising result remains unclear [27, 28]. The inability of trastuzumab to disrupt MAPK and PI3K/Akt signalling is in any case in line with the fact that it is also unable to inactivate ErbB2 in SK-BR-3 cells. Gefitinib and erlotinib did not significantly inhibit PI3K/Akt and MAPK pathways in SK-BR-3 cells, which is coherent with their modest effects on EGFR and ErbB2 phosphorylation (Figure 4 and 5).

In order to investigate which pathway activated by EGFR and ErbB2 was involved in SOCE regulation, we used specific inhibitors of PI3K, Akt, MEK1/2 and JAK1/2. To interfere with PI3K/Akt pathway, we treated SK-BR-3 cells with LY294002 and MK22006 that inhibit PI3K and Akt, respectively. Both inhibitors reduced Ca^2+^ entry after store depletion triggered by thapsigargin (Table 1). As expected, PI3K and Akt inhibitors also decreased the phosphorylation of Akt but had no effect on MAPK pathway (Figure 4 and 5).

Trametinib, an inhibitor of MEK1/2 that are kinases involved in the ERK cascade, did not modify SOCE (Table 1) but significantly reduced ERK1/2 phosphorylation (Figure 5). Ruxolitinib, an inhibitor of JAK1 and JAK2, did not alter SOCE (Table 1).

All those observations showed that SOCE is modulated by the PI3K/Akt pathway but not by the MAPK pathway or the JAK/STAT pathway.

### Effects of SOCE modulation on cell proliferation

**Figure 6:**
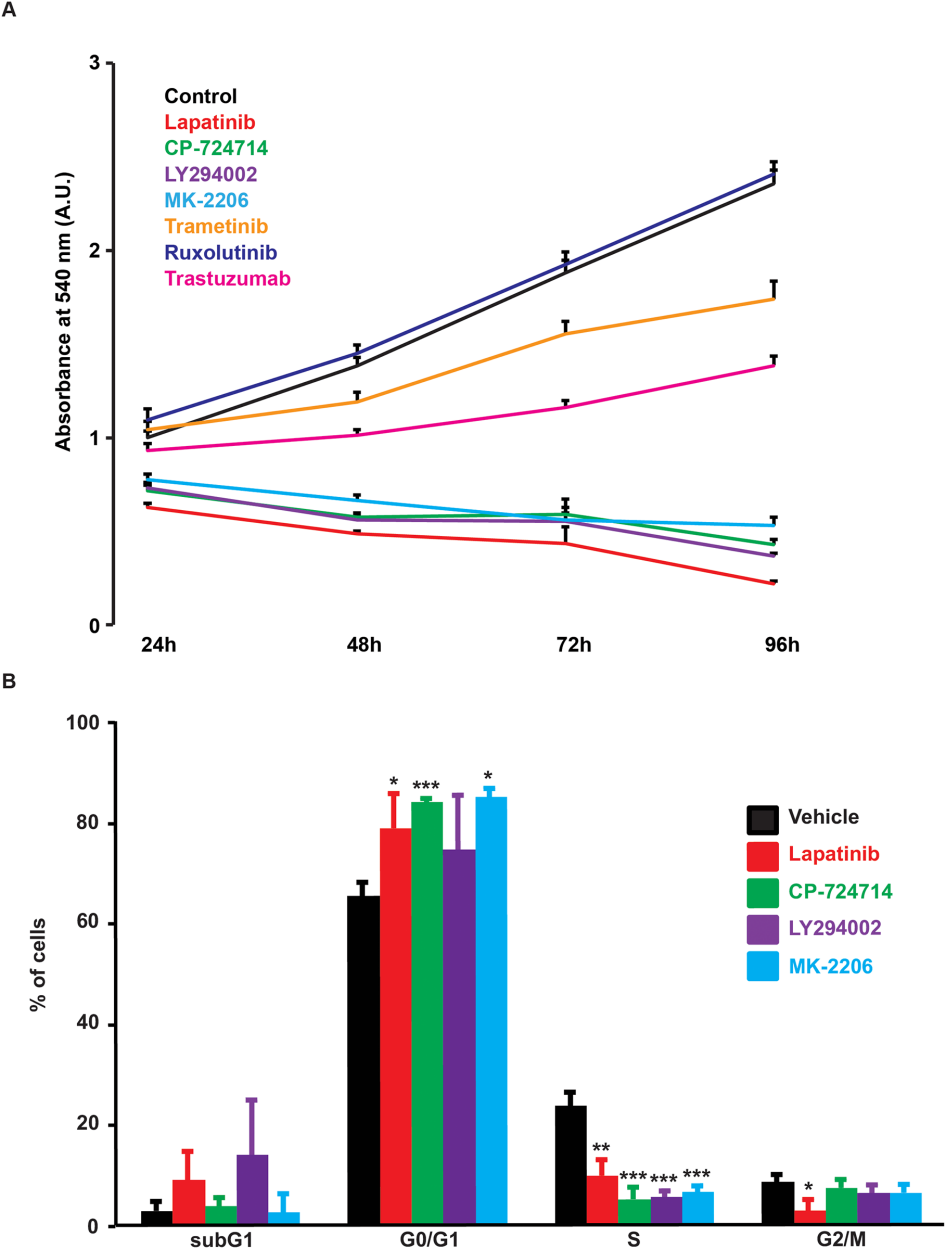
Effects of various kinase inhibitors on SK-BR-3 cells proliferation **(A)** Proliferation curve of SK-BR-3 cells treated with lapatinib 2μM, CP-724714 2μM, LY294002 25μM, MK-2206 1μM, trametinib 10μM, ruxolutinib 1μM, trastuzumab 1μM (or a vehicle) for 24, 48, 72 and 96h in 96-well plates. Cell proliferation was then measured as in Figure 1A. Each treated condition was measured in quadruplicate and compared to the control condition. Results are expressed in percentages as means ± S.E.M. of at least three independent experiments. **(B)** DNA content analysis by PI staining in SK-BR-3 cells treated for 72h with the indicated inhibitor at the same concentrations as in A. Results are expressed as means ± SD, n = 3; ^*^ *P* < 0.05. ^**^ *P* < 0.01, ^***^ *P* < 0.001.

Inhibition of RTK is routinely used to inhibit cancer cell proliferation. As mentioned above, a plethora of studies identified SOCE as a major regulator of cell proliferation. We then analysed whether there was a correlation between inhibition of SOCE and SK-BR-3 proliferation. As shown in Figure 6A, the TKIs that were able to reduce SOCE (i.e. lapatinib and CP-724714) were also able to decrease cell proliferation. In line with our previous observations, inhibition of PI3K/Akt pathway with LY294002 and MK-2206 altered cell proliferation. This means that all inhibitors that reduced Ca^2+^ entry after store depletion were also able to block the proliferation of SK-BR-3 cells. This effect was due to a cell cycle arrest in G0/G1 phase (Figure 6B). Only LY294002 was slightly toxic on SK-BR-3 cells. Obviously, inhibition of SOCE does not constitute the only mechanism of action of agents that target RTKs. It is then expected that some TKIs that had no effect on SOCE amplitude, such as trastuzumab, gefitinib or erlotinib, were able to limit SK-BR-3 proliferation.

### Effects of dual inhibition of EGFR/ErbB2 on SOCE amplitude in a panel of cancer cell lines

**Figure 7:**
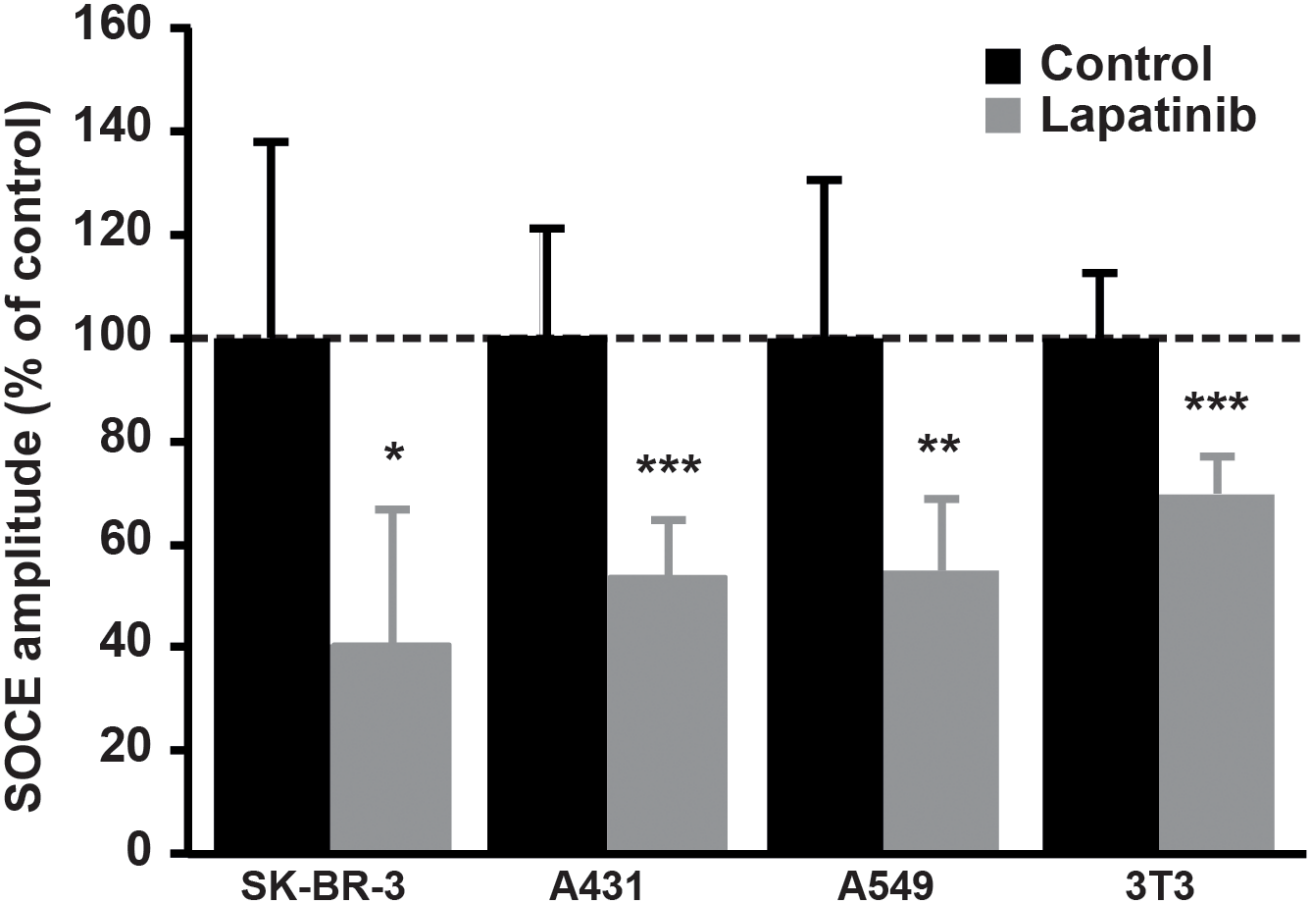
Effect of Lapatinib on SOCE amplitude in a panel of cell lines A549, A431 and NIH-3T3 cells were stained with Fura-2AM (1μM) and SOCE was measured as described in Figure 1. Quantifications of [Ca^2+^]_i_ measured at the peak induced by SOCE after 2μM lapatinib treatment for 10 min are presented as a percentage of the control. Results are expressed as means ± S.D (n=9), Student’s t-test, ^*^ *P* < 0.05. ^**^ *P* < 0.01, ^***^ *P* < 0.001.

**Figure 8:**
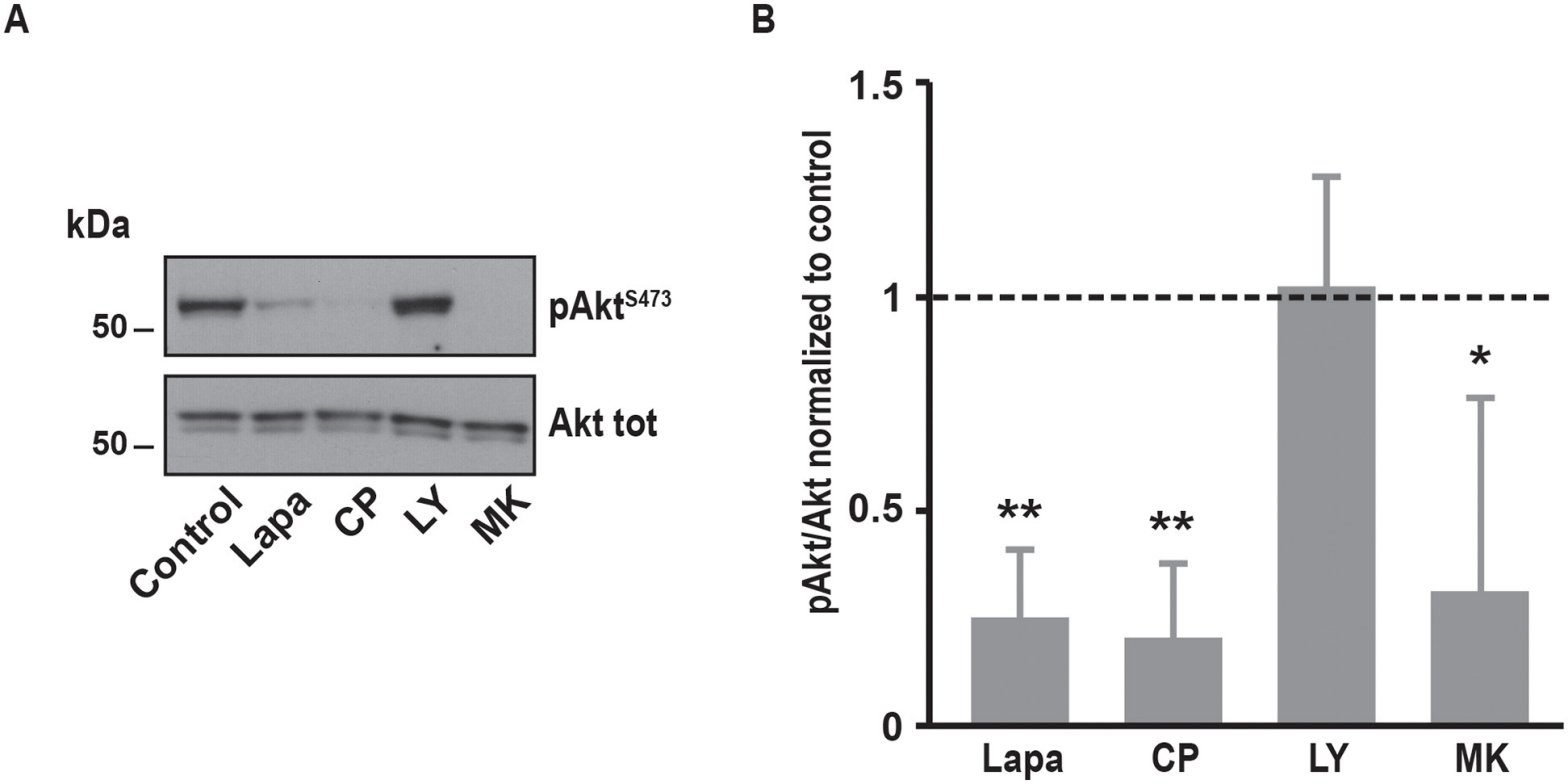
Effects of various inhibitors on Akt phosphorylation in A431 cells **(A)** Immunoblot analysis of phospho-Akt^S473^ and Akt total in A431 cells. The same protocol than in Figure 2 was used, A431 were treated at 37°C for 10 minutes with lapatinib 2μM, CP-724714 2μM, LY294002 25μM, MK-2206 1μM or a vehicle. Immunoblots presented in A are representative of at least three independent experiments. **(B)** Relative quantification of immunoblots presented in A. Akt phosphorylation obtained after treatment was compared to Akt total and then normalized to the control condition. Results are expressed as means ± S.D. of at least three independent experiments, Student’s t-test, ^*^ *P* < 0.05, ^**^ *P* < 0.01.

In order to test whether the effect of lapatinib on SOCE is a general mechanism in cancer cells, we tested a panel of other cell lines derived from a variety of cancer tissues. We observed that, similarly to its effect in SK-BR-3, the dual inhibition of EGFR and ErbB2 by lapatinib was able to decrease SOCE amplitude in non-small cell lung carcinoma cells (i.e. A549), epidermoid carcinoma cells (i.e. A431) and also in an embryonic cell line (i.e. NIH-3T3) (Figure 7). In A431 cells, the PI3K/Akt pathway also mediated this effect since treatment with MK-2206, the specific inhibitor of Akt, diminished SOCE (Table 2). Surprisingly, LY294002 had only minor effect on SOCE in A431 cells. This was correlated with an absence of effect of LY294002 on Akt phosphorylation (Figure 8). In contrast, lapatinib, CP-724714 and MK-2206 dramatically decreased Akt activation in this cell line. Like in SK-BR-3 cells, erlotinib and gefitinib did not reduce SOCE amplitude. This suggests that the interaction between RTK-dependent pathways and SOCE is a general mechanism in proliferating cells.

**Table 2:** Effects of various inhibitors on SOCE amplitude in A431 cancer cells, expressed as percentage of control (mean ± SD)

Inhibitor	SOCE ± SD (% of control)	N	*P* value
Lapatinib 2μM	53.68 ± 11.01	12	<0.001
CP-724714 2μM	86.65 ± 23.31	12	NS
Erlotinib 5μM	110.69 ± 14,08	9	NS
Gefitinib 1μM	117.47 ± 10.81	9	NS
LY294002 25μM	79.36 ± 19.67	11	NS
MK-2206 1μM	76.23 ± 13.50	11	<0.01

SOCE was measured as in Figure 1A.

## DISCUSSION

Since the last two decades, therapies targeting RTKs have become a major weapon in the therapeutic arsenal in oncology and have dramatically improved the outcomes of several types of cancer, such as breast cancers, non-small cell lung carcinomas, head and neck cancers, ovarian cancers, leukemias, … However, as for conventional therapies, the efficacy of targeted therapies is limited in time by the development of resistance mechanisms. A better understanding of the mechanism of action of drugs inhibiting RTKs is then thought to allow the development of novel strategies to circumvent this therapeutic challenge.

Intracellular Ca^2+^ is a major mediator of various processes that are deregulated in cancer cells. In particular, SOCE, which is the main pathway allowing Ca^2+^ entry into the cytosol, regulates cell proliferation, apoptosis, metastasis, angiogenesis and antitumor immunity [29]. Moreover, SOCE is deregulated in several cancer cell lines and molecular players of SOCE could constitute therapeutic targets [30]. However, the role of Ca^2+^ signalling and especially SOCE in the mechanism of action of agents targeting RTKs have been little investigated.

In this study, we showed that inhibition of EGFR and ErbB2 decreased the amplitude of SOCE in breast cancer cell lines (SK-BR-3 cells) and in other cancer or proliferating cells. In several cancer cells overexpressing ErbB2, inhibition of ErbB2 alone by specific inhibitors seems to be impossible since it is well known that ErbB2 is the preferential partners of the ErbB receptors, including EGFR [31]. In line with this observation, CP-724714, a specific inhibitor of ErbB2, decreased EGFR phosphorylation in SK-BR-3 cells. This also suggests that ErbB2 is slightly present in its homodimer conformation and mainly forms heterodimers with EGFR in SK-BR-3 cells. In order to find another specific inhibitor of ErbB2 that might have few effects on EGFR, we also tested trastuzumab, a specific antibody against ErbB2 routinely used in the treatment of advanced breast cancers. However, 10 minutes treatment with trastuzumab had no significant effect on ErbB2 phosphorylation in SK-BR-3 cells. Coherently with this observation, trastuzumab did not reduce the amplitude of SOCE in the same experimental conditions. In contrast, trastuzumab slightly inhibited cell proliferation and reduced ErbB2 activation after chronic exposure.

Specific inhibitors of EGFR in SK-BR-3 cells had only slight effects on EGFR and ErbB2 activation and no effect on SOCE amplitude. This might be the result of the strong expression of ErbB2 in SK-BR-3 cells that constitutively activates EGFR.

Taken together, those results suggest that dual and strong inhibition of both EGFR and ErbB2 is required to decrease SOCE amplitude. Importantly, since dual inhibition of EGFR and ErbB2 also altered ErbB3 and ErbB4 activation, we cannot exclude a potential role of those receptors in the modulation of SOCE.

Since SOCE is involved in cancer cell proliferation, we expected that inhibition of SOCE was correlated with a growth inhibition. This was indeed the case: lapatinib as well as CP-724714 induced cell cycle arrest in G0/G1 phase and dramatically reduced SK-BR-3 cell proliferation. This is in line with our previous results showing that SOCE inhibition in NSCLC cells increased the percentage of cells blocked in G0/G1. Importantly, the effect of dual inhibition of EGFR and ErbB2 by lapatinib on SOCE and cell proliferation was also present in a panel of various cell lines, suggesting that this phenomenon is a general mechanism in proliferating cells.

We then investigated which downstream signalling pathways were involved in SOCE regulation. It has been recently shown that STIM1 was directly phosphorylated by ERK1/2 in HEK293 cells after stimulation by IGF-1 [32]. In our study, inhibition of MAPK pathway by trametinib had no effect on SOCE amplitude, demonstrating that, at least in SK-BR-3 cells, the function of molecular mediators of SOCE is not modulated by ERK1/2. Consistently with its absence of effect on SOCE, trametinib had no significant effect on cell proliferation.

We also tested the potential role the PI3K/Akt pathway in SOCE modulation. We used two different inhibitors of this pathway. LY294002 is a competitive inhibitor of the ATP-binding site of the PI3K catalytic subunit [33]. MK-2206 is an allosteric inhibitor of Akt that is currently under investigation for the treatment of several types of cancer, notably breast cancer [34]. Both inhibitors dramatically inhibited SOCE amplitude and cell proliferation. In line with this observation, a recent study has shown that Akt activation, in response to EGF stimulation, phosphorylated STIM1, this triggering SOCE [35]. In contrast to trametinib, inhibition of PI3K/Akt pathway reduced SK-BR-3 cell growth.

In conclusion, our results show that some TKIs are able to negatively regulate SOCE. As this process is involved in cancer development and progression, its inhibition by TKIs could contribute to their anticancer action. Furthermore, since SOCE mediators such as STIM1 and ORAI1 are elevated in cancer cells and contribute to cancer aggressiveness, the exacerbation of SOCE might constitute a mechanism of resistance against TKI therapies.

## MATERIALS AND METHODS

### Cell culture and reagents

SK-BR-3, A431, NIH-3T3 and A549 cell lines (American Type Culture Collection) were grown in DMEM or DMEM-F12 medium supplemented with 10% FCS at 37 °C in an humidified atmosphere of 5% CO_2_. Cells were cultured up to passage 30. Thapsigargin (Sigma-Aldrich) is used at 1 μm. Lapatinib (2μM), CP-724714 (2μM), MK-2206 (1μM) and trametinib (10μM) were purchased from Selleckchem. LY294002 (25μM), gefitinib (1μM), and erlotinib (5μM) were provided by Tocris, ruxolitinib (1μM) by VWR and trastuzumab (1μM) by Roche.

### siRNA transfection

Depletion of STIM1 was achieved by using a pool of 4 siRNAs (called as siSTIM1) targeting 4 different sequences of human STIM1 mRNA. siSTIM1 (Catalog #L-011785-00) as well as the non-silencing control pool of siRNAs (siUNR, Catalog #D-001810-10) were purchased from Thermo Fisher Scientific (Lafayette, CO, USA). SK-BR-3 cells were transfected using DharmaFECT reagent according to the manufacturer’s instructions (Thermo Fisher Scientific). Twenty four hours later, cells were plated on 6-well plates or on 10cm-diameter Petri dishes. Cells were analyzed 96 hours after transfection.

### Cytosolic free Ca^2+^ measurements

SK-BR-3, A431, A549 and NIH 3T3 cells were plated on 22-mm round glass coverslips (2×10^5^ cells/well, 6 well plates) and grown during five days. Cells were incubated with 1 μm of Fura-2/AM (Invitrogen) in Krebs-HEPES buffer (11.5 mM HEPES, 120 mM NaCl, 6 mM KCl, 1.8 mM CaCl_2_, 1.2 mM MgCl_2_, 10 mM d-glucose, pH 7.6) for 1h15 at room temperature. Coverslips were then washed promptly and mounted in a heated (37 °C) microscope chamber. Cells were alternately excited at 340 and 380 nm using a Lambda DG-4 Ultra High Speed Wavelength Switcher (Sutter Instrument) coupled to a Zeiss Axiovert 200M inverted microscope. Images were acquired with a Zeiss Axiocam camera coupled to a 510 nm emission filter and analysed with Axiovision software. Calcium concentration was evaluated from the ratio of fluorescence emission intensities excited at the two wavelengths using the Grynkiewicz equation [36].

### Immunoblotting

To mimic experimental conditions used for Ca^2+^ measurements, cells were cultured for 1h30 in the absence of FCS before treatment with each inhibitor.

Two different protocols were used to lyse the cells depending on the cell type. SK-BR-3 cells were fractionated to separate cytosolic fraction and membrane fraction. A total lysis was used for the other cell types.

A membrane protein extraction kit (Mem-PER plus kit, Thermo Scientific) was used to extract SK-BR-3 proteins. Cells were processed according to the manufacturer’s recommendations. SK-BR-3 were harvested by scraping in PBS, rinsed twice with ice-cold PBS, and re-suspended in the permeabilisation buffer. After centrifugation, the supernatant containing cytosolic protein was transferred in a new tube and the pellet, containing membrane proteins, was diluted in the solubilisation buffer. Permeabilisation and solubilisation buffers were supplemented with sodium orthovanadate (1mM), NaF (50mM), NaPPi (10mM), and protease inhibitor cocktail (Sigma).

Like for SK-BR-3 lysis, A431 were harvested by scraping in PBS, rinsed twice with ice-cold PBS, and then re-suspended in lysis buffer (Cell Lysis Buffer, Abcam) containing 0.216% Beta glycerophosphate, 0.19% Sodium orthovanadate, 0.001% leupeptin, 0.38% EGTA, 10% Triton-X-100, 3.15% Tris HCl, 8.8% Sodium chloride, 0.29% Sodium EDTA and 1.12% Sodium pyrophosphate decahydrate.

In both cases extracts were diluted in a mix of LDS sample buffer and sample reducing Agent (NuPAGE^®^, Invitrogen) and heated at 95°C for 3 min. Samples were electrophoresed on 7% or 10% SDS-polyacrylamide gels (Invitrogen) and transferred onto nitrocellulose (Bio-Rad) or PVDF membranes (Millipore). The blots were saturated with TBS-T buffer (0.02 M Tris(hydroxymethyl)aminomethane, 0.138 M NaCl, 0.05% Tween 20, pH 7.6), containing 5% BSA or milk for 1 h at room temperature, incubated overnight at 4 °C with primary antibodies: anti-EGFR, anti-phospho-EGFR^Y1068^, anti-ErbB2, anti-phospho-ErbB2^Y1248^, anti-ErbB3, anti-phospho-ErbB3^Y1289^, anti-phospho-ErbB4^Y984^, anti-Akt, anti-phospho-Akt^S473^, anti-ERK1/2, anti-phospho-ERK1/2, GAPDH (1:1000, Cell Signaling Technology), and anti-β-actin (1:10,000, Sigma-Aldrich). After incubation with appropriate secondary antibodies coupled to peroxidase, peroxidase activity was detected with ECL (WESTAR C2.0, CYANAGEN) on ECL hyperfilm. Immunoblots were quantified using the ImageJ program. All lanes separated by a thin line have been cropped from the same immunoblot.

### Proliferation assay and cell cycle analysis

Cell proliferation was determined 72 hours after incubation in the presence of different inhibitors using the *In Vitro* Toxicology Assay Kit Sulforhodamine B based (Sigma-Aldrich). For viability assays, SK-BR-3 cells were plated at 1000 cells/well in 96-well plates. Cells were allowed to grow in a medium without inhibitors during 24h, then they were treated with the appropriate concentration of inhibitor. All the conditions were done in quadruplicate and repeated independently at least three times. Cells were fixed, washed and incubated with sulforhodamine B according to the manufacturer recommendations. 96-well plates were then red at 540 and 690 nm.

Measurement of DNA content was performed by permeabilizing fixed cells with 0.01% triton X-100 and subsequent staining by 50 μg/ml propidium iodide (PI). Cytofluorometric analyses were performed on a FACSCalibur equipped with CellQuest Pro software (Becton Dickinson). Cell cycle analysis was performed with the FlowJo software (FlowJo LLC).

### Statistical analysis

Data are presented as means ± SD. Student’s *t*-test and analysis of variance were used to determine statistical significance when appropriate.
